# Canis mtDNA HV1 database: a web-based tool for collecting and surveying Canis mtDNA HV1 haplotype in public database

**DOI:** 10.1186/s12863-017-0528-0

**Published:** 2017-06-26

**Authors:** Quan Ke Thai, Dung Anh Chung, Hoang-Dung Tran

**Affiliations:** 1 Saigon University, 273 An Duong Vuong street, District 5, Ho Chi Minh city, Vietnam; 2 Institute of Agricultural science for Southern Vietnam, 121 Nguyen Binh Khiem street, District 1, Ho Chi Minh city, Vietnam; 3 Nguyen Tat Thanh University, 300A Nguyen Tat Thanh street, District 4, Ho Chi Minh city, Vietnam

**Keywords:** Control region, *Canis lupus familiaris*, Database, Haplotype identifier, HV1 haplotype

## Abstract

**Background:**

Canine and wolf mitochondrial DNA haplotypes, which can be used for forensic or phylogenetic analyses, have been defined in various schemes depending on the region analyzed. In recent studies, the 582 bp fragment of the HV1 region is most commonly used. 317 different canine HV1 haplotypes have been reported in the rapidly growing public database GenBank. These reported haplotypes contain several inconsistencies in their haplotype information. To overcome this issue, we have developed a Canis mtDNA HV1 database. This database collects data on the HV1 582 bp region in dog mitochondrial DNA from the GenBank to screen and correct the inconsistencies. It also supports users in detection of new novel mutation profiles and assignment of new haplotypes.

**Description:**

The Canis mtDNA HV1 database (CHD) contains 5567 nucleotide entries originating from 15 subspecies in the species *Canis lupus*. Of these entries, 3646 were haplotypes and grouped into 804 distinct sequences. 319 sequences were recognized as previously assigned haplotypes, while the remaining 485 sequences had new mutation profiles and were marked as new haplotype candidates awaiting further analysis for haplotype assignment. Of the 3646 nucleotide entries, only 414 were annotated with correct haplotype information, while 3232 had insufficient or lacked haplotype information and were corrected or modified before storing in the CHD.

The CHD can be accessed at http://chd.vnbiology.com. It provides sequences, haplotype information, and a web-based tool for mtDNA HV1 haplotyping. The CHD is updated monthly and supplies all data for download.

**Conclusions:**

The Canis mtDNA HV1 database contains information about canine mitochondrial DNA HV1 sequences with reconciled annotation. It serves as a tool for detection of inconsistencies in GenBank and helps identifying new HV1 haplotypes. Thus, it supports the scientific community in naming new HV1 haplotypes and to reconcile existing annotation of HV1 582 bp sequences.

**Electronic supplementary material:**

The online version of this article (doi:10.1186/s12863-017-0528-0) contains supplementary material, which is available to authorized users.

## Background

Mitochondrial DNA (mtDNA) has been demonstrated to be a valuable tool for genetic characterization of animal samples [1–3]. Many studies had been implemented focusing on these DNA sequences to evaluate the genetic diversity of dog populations [4, 5], determine the evolution relationship among them [6], or trace the origin of a dog breed [7–9]. Although different regions in the mtDNA were used, such as 16S rRNA gene [10], cytochrome oxidase subunit 1 (COI) gene [6] or the whole mtDNA genome [11], the 582 bp region (from nucleotide 15,458 to 16,039) of hypervariable region 1 (HV1) were widely exploited to study the relationship among different dog breeds, and consequently, infer the origin of a dog breed [4, 7, 12].

The D-loop (control region) of dog mtDNA consists of two hypervariable regions (HV1 and HV2) which are separated by a region of variable number tandem repeat [13]. HV1 is the most variable part of dog mtDNA while the sequence variation rate of HV2 is similar to those of other regions in mtDNA [14]. Since the full-length sequence of a domestic dog (*Canis lupus familiaris*) mitochondrial DNA were firstly reported [13], the number of HV1 nucleotide sequences has increased rapidly in the public database GenBank [15]. A BLAST search using the reference sequence (GenBank accession U96639.2 [13]) as query sequence, identified 45,415 nucleotide sequences originating from 864 different species and subspecies (data not shown). GenBank, which was developed by National Center for Biotechnology Information – USA (NCBI), is open for submission of new nucleotide sequences with some checks for vector contamination, translation of coding regions, correct taxonomy and correct bibliographic citations [15]. Invalidated sequence annotations may therefore result in errors and inconsistencies which needs a tool for correcting and reconciling [16, 17].

In GenBank, dog mtDNA sequences were not usually annotated with haplotype information and when it existed it was expressed using different systems because of different research goals. Annotated haplotype information can be deduced from a small region (60 bp) [3], from the 582 bp region [11], from the 660 bp region [18] in the mtDNA HV1, or from the combination of the 582 bp region and the mtDNA HV2 [19]… in which, the 582 bp region was used in most recent studies [9, 11, 20–22]. According to this system, all haplotypes of dog mtDNA belongs to six phylogenetic groups named from A to F. A haplotype was named by the corresponding haplogroup followed by an Arabic numeral (e.g., B15) [20]. The nomenclature for dog mtDNA D-loop was proposed using this A-F system [23].

The Canis mtDNA HV1 database (CHD) was built up to collect 582 bp sequences of dog mtDNA HV1 region from public database GenBank to facilitate the screening, reconciliation or correction of inconsistencies and errors as well as to support users in detection of new novel mutation profiles and assignment of new haplotypes.

## Construction and content

### Construction

#### Development and construction of CHD

The 582 bp HV1 fragment of the *Canis lupus familiaris* mitochondrion (GenBank accession: U96639.2) was used as a seed sequence for building up the CHD. A BLAST search [24] was performed against the NCBI nucleotide database [25] without filtering of low complexity regions, and with a low E-value threshold (10e-94) to prevent the occurrence of low similar sequences in the BLAST results. The filter concerning taxonomy was also set to limit sequences not originating from the species *Canis lupus*. For each hit in the BLAST result, the accession number was extracted and the complete GenBank entry was downloaded from the NCBI nucleotide database. Information on sequence, definition, source organism, annotations related to the 582 bp fragment was extracted from the entry and parsed by an automated retrieval system into an in-house developed relational database system.

The CHD is managed by MySQL, running on Linux system. Scripts for the back-end and query interface were developed in Perl. For the web-based query interface, the Apache web server [26] is used. The data will be updated regularly by an automated Perl script basing on the latest released version of the GenBank.

#### Multisequence alignment, nucleotide numbering and mutation illustration

Each nucleotide sequence in the CHD was aligned with reference sequence (GenBank accession: U96639.2) [13] using ClustalW [27]. The alignment result was adjusted using the proposed strategy [23] to achieve right position of inserted and deleted nucleotides. Individual nucleotide in the sequence were numbered according to the standard scheme suggested by Pereira et al. [23]. A change concerning one nucleotide is illustrated with the format XnumY where num is the numbered position, X is the nucleotide at the position num on the reference sequence and Y is the corresponding nucleotide on the examined sequence; the missing nucleotide (a gap) would be a “-”. For example, T15639G is the substitution of a thymine at position 15,639 by a guanine while T15465- is the deletion of thymine at position 15,465 and −15,535.1C -15,535.2C denotes the insertion of two nucleotide cytosine after the nucleotide at position 15,535. A set of nucleotide substitutions, deletions, and insertions occurring in the 582 bp region of each sequence was recorded and considered as mutation profile of the sequence.

#### Haplotype identification of mtDNA D-loop HV1 sequence

Nucleotide sequences representing assigned haplotypes were aligned for identification of substitution motif of each haplogroup (Table 1). These substitution motifs will then be used to classify a haplotype with new mutation profile into a haplogroup. On the sample of 50 random sequences, this method of haplogrouping worked well giving the similar result with the haplogrouping using phylogenetic tree (Additional file 1: Table S1).

**Table 1:** Substitution motifs of haplogroups

Haplogroup	Substitution motif
B	C15526, T15612, C15632, T15639, G15652, T15800, C15814 C15955
C	C15508, C15526, T15639, T15650, T15800, C15912, C15955
D	T15625, C15632, T15636, T15639, T15800, C15814, T15815, G15848, C15912, C15959
E	C15526, A15553, T15639, G15652, T15800, C15814, C15912, G15938
F	A15490, T15523, T15611, A15627, T15628, T15639, G15652, T15800, C15814, C15912

**Table 2:** List of species found in CHD

	Species/subspecies	Number of sequences
1	*Canis lupus*	*990*
2	*Canis lupus campestris*	*1*
3	*Canis lupus chanco*	*41*
4	*Canis lupus desertorum*	*1*
5	*Canis lupus dingo*	*28*
6	*Canis lupus familiaris*	*4464*
7	*Canis lupus hattai*	*1*
8	*Canis lupus variabilis*	*1*
9	*Canis lupus hodophilax*	*10*
10	*Canis lupus labradorius*	*1*
11	*Canis lupus laniger*	*2*
12	*Canis lupus lupaster*	*10*
13	*Canis lupus lupus*	*3*
14	*Canis lupus pallipes*	*5*
15	*Canis lupus signatus*	*9*

**Table 3:** Entries with inconsistent annotations

GenBank acc.	Mutation profile	Haplotype		Inconsistency
		according to GenBank	recognized by CHD	
KM262649.1	A15627G T15639A C15814T A15931- C15959T T16025C A16033G	A228	A140 (A15627G T15639A C15814T A15931- C15959T T16025C A16033G)	assignment of a new haplotype for an assigned haplotype
JF342817.1	A15653G C15814T C15955T	A17 (T15620C A15627G T15639A C15814T C15955T)	new haplotype A (A15653G C15814T C15955T)	Wrong identification for a new haplotype
JF342836.1	A15553G T15639A C15814T A15931-)	A171 (T15639A C15814T G15848A T16025C)	A246 (A15553G T15639A C15814T A15931-)	Wrong identification for an assigned haplotype
KJ637102.1	C15483T A15627G T15639A C15814T C15912T	haplotype Be36_2	A1 (C15483T A15627G T15639A C15814T C15912T)	Annotated with other haplotyping system

The mutation profile of each sequence in CHD was identified and matched against mutation profiles of assigned haplotypes to identify whether the respective mutation profile is identical to an already assigned haplotype. If the mutation profiles were identical, the haplotype was defined accordingly (e.g. “A117”). Otherwise it would undergo a further comparison with substitution motif of haplogroups to be assigned as a new member of the matched group with the format (haplogroup)n(number), where (haplogroup) is A, B, C, D, E or F, n stands for “new” and (number) is the order number, e.g. An1 for the first new haplotype A, Bn15 for the 15th new haplotype B. The new mutation profile was also stored in the CHD.

Previously published sequences, which were haplotyped in other systems, were checked and classified into the current system (using Latin alphabet - from A to F) by the actual mutation profiles.

#### Reconciliation of data inconsistencies

Assigned haplotypes and the haplotype of a sequence would be identified by following rules: (1) The sequence which was firstly used to report a haplotype would be the standard sequence for that haplotype. (2) If two assigned haplotypes have the same mutation profile, the later (newer) will be eliminated. (3) If a sequence was annotated as an assigned haplotype but it is not identical to the standard sequence of that haplotype, the annotation is considered as wrong and its haplotype is defined according to its mutation profile. In CHD, a sequence was classified into a certain haplotype or haplogroup based on its mutation profile regardless of the content of its original definition or annotation.

### Content

#### Data content of the CHD

By BLAST searching against GenBank nucleotide database, 5567 entries were collected and stored in CHD. Of these, the 1921 entries containing partial segments of 582 bp region were not subjected to the haplotyping process. The 3646 remaining entries were grouped into 804 distinct sequences based on the 582 bp region. Of these 804 distinct sequences, the 319 sequences were recognized as assigned haplotypes, and the 485 remaining sequences with new mutation profiles were marked as new haplotype candidates awaiting further analysis for haplotype assignment.

Most of nucleotide sequences collected and stored in CHD originate from *Canis lupus familiaris* (4464 sequences) while remaining sequences are from the species *Canis lupus* and its 13 other subspecies (Table 2).

In the 319 assigned haplotypes recorded in CHD, there are 234 members of the haplogroup A, 49 members of the haplogroup B, 20 of the haplogroup C, 9 of the haplogroup D, 4 of the haplogroup E and 3 of the haplogroup F. Haplotypes in each haplogroup were numbered consecutively, however, there are some missing links in the chain. In haplogroup A, the haplotype A37, A108, A118, A191, A211, A228-A244, A247, A250, A252, A253, A255–257, A259, A260, A263-A272 are missing, although the haplotype with highest number in this haplogroup is A275. In haplogroup B, the haplotype B55 is recorded but the haplotypes B31, B42, B43, B51, B53 and B54 are missing. Similarly, the haplotype C9 and D9 are missing in haplogroup C and haplogroup D, respectively.

#### Analysis of polymorphic sites and nucleotide substitutions

**Fig. 1:**
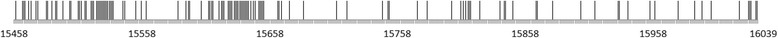
Polymorphic sites in the 582 bp region

In assigned haplotypes, there are totally 136 polymorphic sites in the 582 bp region spreading over the sequence, in which, 92 sites (67.6%) are in the first 200 bp (Fig. 1).

Single insertion can be found at nucleotide positions 15,464, 15,534 in some haplotypes. Only in the haplotype A133, there is an 11-nucleotide insertion (CCCCCTCCCCT) after the nucleotide at position 15,535. At some positions such as 15,465, 15,525, only the deletion occurred while at some positions such as 15,526, 15,673, there are transitions in some haplotypes and deletions in other haplotypes. The transversion rarely occurred in this region. Only 11 positions (8.08% of polymorphic sites) were found with transversion occurred, of these, 5 positions (15,458, 15,631, 15,638, 15,639, 15,651) were found with transversion in some haplotypes and transition in others.

#### Data inconsistencies

In the 5567 mtDNA entries collected from GenBank, 3646 entries with full-length sequence of the 582 bp region underwent further analysis for the accuracy of annotation. Only 414 entries were correctly haplotyped and annotated with haplotype information using A-F system, while 1359 entries were annotated with wrong haplotype identification or with the haplotyping information using other systems. For example, the nucleotide sequence with GenBank accession JF342836.1 has 3 substitutions and one deletion (A15553G T15639A C15814T A15931-), therefore, it should be haplotyped as “A246”, but it is actually annotated as “A171” which was defined by 4 substitutions (T15639A C15814T G15848A T16025C) (Table 3). The remaining 1873 entries did not contain any information about haplotype. The lack of haplotype information and inconsistencies derived from the GenBank were modified or corrected in CHD.

## Utility

All haplotyped sequences were grouped into 319 assigned haplotypes which in turn were grouped into 6 haplogroups A, B, C, D, E and F. Every haplotype was presented together with its corresponding mutation profile. The nucleotide sequences were labeled by GenBank accession numbers and linked to the GenBank database. The mutation table provides an overview to the nucleotide substitutions of all assigned haplotypes. Polymorphic sites occurring along the 582 bp region were shown with the nucleotide substitutions of each haplotype. By hovering the cursor over a nucleotide in the table, the corresponding haplotype, nucleotide position and the reference nucleotide will be displayed. For example, “A11…T15639” displayed when hovering a cell with “A” means that a thymine at position 15,639 were substituted by an adenine in haplotype A11. Haplotype of a query nucleotide sequence can be identified by “haplotype identifier”. In case of a new haplotype, the mutation profile and the nearest assigned haplotype will be shown. The CHD is accessible at http://chd.vnbiology.com by a Javascript-enabled web browser.

## Discussion

### Data content of the CHD

In CHD, although the whole sequence of each entry was aligned with reference sequence, only the 582 bp region was analyzed for haplotyping, then different sequences with 100% identical in 582 bp region would be classified into the same haplotype. By systematic analysis of nucleotide sequences in CHD, 485,582-bp sequences were temporarily identified as new haplotypes. Of which, 391 sequences are in the haplogroup A, 56 sequences are in the haplogroup B, 28 sequences are in the haplogroup C, 3 sequences are in the haplogroup D, 5 sequences are in the haplogroup E and 2 sequences are in the haplogroup F. These new haplotypes will undergo a further analysis for haplotype assignment.

In published publications, a substitution, for simplicity reasons, is usually illustrated in the format like 15,627 or 15639^T/A^, where number is the position of the nucleotide; the number without superscript denotes a transition while the number with superscript denotes a transversion [23]. In the case of transversion, the readers know the original nucleotide of the substitution (thymine to adenine in the above example) but in the case of transition, there is no information about the original and the substituted nucleotide. The deletion are marked with superscript “del” while the insertion is presented as 15,534.1C or 15,534.2C for one or two cytosines inserted after nucleotide at position 15,534 [23]. In this format, the number after a decimal point is the amount of nucleotides inserted, so the format is only suitable for homopolymeric tract, and cannot be applied in the case of heteropolymeric tract, for example, in haplotype A133 with the CCCCCTCCCCT insertion. Many different formats would make it difficult to do systematic analysis. In CHD, all kinds of nucleotide changing (including indels) are illustrated with the same format “XnumY” which facilitates the automatic analysis, and would be more user-friendly.

Most of haplotypes in haplogroup A differed from the nearest haplotype by 1 or 2 nucleotides, while a few haplotypes differed from the nearest haplotype by 4 nucleotides. One exception is the haplotype A133 with the CCCCCTCCCCT insertion, which results in the isolation of this haplotype from other haplotypes in the haplogroup. The nearest distance between this haplotype and another is 12 substitutions in the case of A11. This 11-mer insertion, which is seen only in one haplotype A133, can cause the doubt of the accuracy of the data. Interestingly, although this insertion presents in one haplotype, it can be seen in two different sequences reported by two different authors. Firstly reported by Pang et al. in 2009 [11], this insertion had not been seen in another sequence until 2015, when Duleba et al. published a complete mtDNA sequence originated from *Canis lupus familiaris* (GenBank accession KM061501.1). It is noteworthy that neither haplotype information nor the CCCCCTCCCCT insertion was mentioned in this GenBank entry. The insertion had been only discovered during the analysis process of CHD and the 582 bp region of this sequence is 100% identical with the haplotype A133. Hence, there was a strong confirmation for the existence of the CCCCCTCCCCT insertion in a certain *Canis lupus familiaris* mtDNA.

**Fig. 2:**
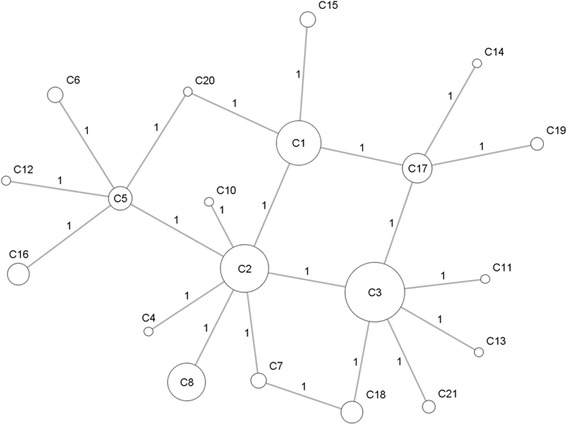
Network formed by haplotypes in haplogroup C. The number by a link denotes the number of nucleotide differences between two haplotypes. Size of a node indicates the commonness of the corresponding haplotype in CHD

During the evolution, mtDNA accumulates mutations gradually. This can be confirmed via network of haplotypes in each haplogroup with mutations as links. Especially, haplotypes in haplogroup C form a perfect network, in which, two haplotypes differ from each other by only one nucleotide (Fig. 2). In networks of other haplogroups, two adjacent haplotypes differing from each other by more than one nucleotide can be seen, which suggests that the intermediate haplotypes can be discovered in the future.

#### Data inconsistencies and reconciliation

Failing in substitution identification is a cause of data inconsistency [28, 29]. If this error is submitted to GenBank, it will affect users who usually just fetch data from GenBank without verification. Another common cause is the haplotype numbering. Probably, the haplotype numbering is spontaneously implemented by a researcher or a research group, not in the agreement of the research community. It is supposed that when a sequence with new mutation profile is defined, the researcher will determine the highest haplotype number in the corresponding haplogroup in GenBank and simply names the new haplotype with the following number. The fact showed that there could be two mistakes occurring during this process: being wrong in haplotype identification or skipping the haplotype number. This leads to the need of the availability of a reasonable tool for surveillance of 582 bp sequences and new haplotypes. Although CHD can identify a haplotype of a certain 582 bp sequence, the assignment of a new haplotype should be confirmed and announced by an experienced researcher in the field.

## Conclusion

The Canis mtDNA HV1 database (CHD) was established to collect 582 bp sequences originated from *Canis lupus* and its subspecies in GenBank. Besides 414 entries annotated with correct haplotype information, 1359 entries with inconsistent annotations were revealed and reconciled. Moreover, haplotype information was provided to entries, which lacked this information. The CHD thus supports the scientific community to name new haplotype and to reconcile existing annotation of 582 bp sequences.

## Additional file

Additional file 1: 50 random sequences were haplogrouped using the Haplotype identifier and the phylogenetic tree. The data shows the similarity between two different methods in haplogrouping of 50 random sequences. Both the phylogenetic tree and the Haplotype identifier can exactly classify a sequence into a certain haplogroup. The Haplotype identifier can even recognize a known haplotype. (DOC 66 kb)

## Abbreviations

CHD: Canis mtDNA HV1 database
mtDNA: mitochondrial DNA
