# Supplementary material for: Canis mtDNA HV1 database: a web-based tool for collecting and surveying Canis mtDNA HV1 haplotype in public database
Source: BMC Genet. 2017 Jun 26;18:60. doi: 10.1186/s12863-017-0528-0 (PMC5485557; doi:10.1186/s12863-017-0528-0)
Supplement: Additional file 1: — 50 random sequences were haplogrouped using the Haplotype identifier and the phylogenetic tree. The data shows the similarity between two different methods in haplogrouping of 50 random sequences. Both the phylogenetic tree and the Haplotype identifier can exactly classify a sequence into a certain haplogroup. The Haplotype identifier can even recognize a known haplotype. (DOC 66 kb) [file 12863_2017_528_MOESM1_ESM.doc]

Table S1: 50 random sequences were haplogrouped using the Haplotype identifier and the phylogenetic tree.

Two methods successfully haplogrouped all sequences. While the phylogenetic tree can only classify a sequence into a haplogroup, the Haplotype identifier can even recognize a known haplotype. In the case of a sequence harbouring a new mutation profile, the Haplotype identifier classify it into a haplogroup by matching the substitution motif of the corresponding haplogroup and the mutation profile of the sequence.

|  | **GenBank accession** | **Haplotype identifier** | **Phylogenetic tree** |
| --- | --- | --- | --- |
| 1 | KM061511.1 | A1 | Haplogroup A |
| 2 | AB605501.1 | A1 | Haplogroup A |
| 3 | KJ637099.1 | A5 | Haplogroup A |
| 4 | EU223487.1 | A11 | Haplogroup A |
| 5 | KM061549.1 | A11 | Haplogroup A |
| 6 | EF122414.1 | A16 | Haplogroup A |
| 7 | JF342851.1 | A17 | Haplogroup A |
| 8 | D83617.1 | A17 | Haplogroup A |
| 9 | AF531669.1 | A17 | Haplogroup A |
| 10 | JF342812.1 | A18 | Haplogroup A |
| 11 | HQ997449.1 | A18 | Haplogroup A |
| 12 | EU223687.1 | A20 | Haplogroup A |
| 13 | EU223655.1 | A26 | Haplogroup A |
| 14 | AY240089.1 | A29 | Haplogroup A |
| 15 | KF002332.1 | A30 | Haplogroup A |
| 16 | AF531684.1 | A32 | Haplogroup A |
| 17 | AY240100.1 | A33 | Haplogroup A |
| 18 | EU816489.1 | A117 | Haplogroup A |
| 19 | KF002304.1 | A176 | Haplogroup A |
| 20 | HQ452453.1 | A222 | Haplogroup A |
| 21 | KU290672.1 | Haplogroup A | Haplogroup A |
| 22 | KU290445.1 | Haplogroup A | Haplogroup A |
| 23 | AB605507.1 | Haplogroup A | Haplogroup A |
| 24 | KJ139357.1 | Haplogroup A | Haplogroup A |
| 25 | KM201268.1 | Haplogroup A | Haplogroup A |
| 26 | KF574036.1 | Haplogroup A | Haplogroup A |
| 27 | KU290692.1 | Haplogroup A | Haplogroup A |
| 28 | JF342842.1 | B1 | Haplogroup B |
| 29 | EU223476.1 | B1 | Haplogroup B |
| 30 | AF531723.1 | B2 | Haplogroup B |
| 31 | EU816542.1 | B25 | Haplogroup B |
| 32 | HM560915.1 | B36 | Haplogroup B |
| 33 | HQ452475.1 | B40 | Haplogroup B |
| 34 | JF342826.1 | B41 | Haplogroup B |
| 35 | HM560920.1 | Haplogroup B | Haplogroup B |
| 36 | EU223589.1 | Haplogroup B | Haplogroup B |
| 37 | JF342908.2 | Haplogroup B | Haplogroup B |
| 38 | KF661056.1 | Haplogroup B | Haplogroup B |
| 39 | EU223397.1 | C3 | Haplogroup C |
| 40 | KM201262.1 | C5 | Haplogroup C |
| 41 | EF380220.1 | C7 | Haplogroup C |
| 42 | EU816553.1 | C13 | Haplogroup C |
| 43 | KJ637138.1 | Haplogroup C | Haplogroup C |
| 44 | KF002323.1 | D1 | Haplogroup D |
| 45 | AF098138.1 | D1 | Haplogroup D |
| 46 | KF574016.1 | D6 | Haplogroup D |
| 47 | D83632.1 | E1 | Haplogroup E |
| 48 | AB480744.1 | Haplogroup E | Haplogroup E |
| 49 | EF380227.1 | Haplogroup E | Haplogroup E |
| 50 | AB499822.1 | Haplogroup F | Haplogroup F |
